# Sexual health in patients with rheumatoid arthritis and the association between physical fitness and sexual function: a cross-sectional study

**DOI:** 10.1007/s00296-018-4023-3

**Published:** 2018-04-11

**Authors:** Thomas Ernst Dorner, Carolin Berner, Sandra Haider, Igor Grabovac, Thomas Lamprecht, Karl Heinrich Fenzl, Ludwig Erlacher

**Affiliations:** 1 Department of Social and Preventive Medicine, Centre for Public Health, Medical University of Vienna, Kinderspitalgasse 15/I, 1090 Vienna, Austria; 2 Karl Landsteiner Institute for Autoimmune Diseases and Rheumatology, Vienna, Austria; 3 Department of Rheumatology and Osteology, Kaiser Franz Josef Hospital, Sozialmedizinisches Zentrum-Süd, Vienna, Austria

**Keywords:** Sexual health, Physical fitness, Muscle strength, physical performance

## Abstract

The aim of this study was to examine sexual health in patients with rheumatoid arthritis (RA), and to analyse factors associated with sexual health with a focus on physical fitness. One hundred RA patients aged between 18 and 65 years were included in a cross-sectional study. Handgrip strength and knee extensor strength were measured with a dynamometer, and physical performance with the Short Physical Performance Battery (SPPB). Fifty-four patients, mean age 47.8 (SD 10.6) years, 61% female, answered a questionnaire about sexual health. Fifty-seven percent reported, at least, sometimes having difficulty with sexual intercourse (27.8% due to joint stiffness, 24.1% due to fatigue, 18.5% due to pain). Handgrip strength and knee extensor strength significantly correlated with the desire to engage in sexual intercourse, frequency of sexual contact and satisfaction with overall sex life. The SPPB total score correlated with satisfaction with overall sex life, and the SPPB repeated chair stands test with the desire to have sexual intercourse and satisfaction with overall sex life. After adjusting for age, gender, disease activity, comorbidity, co-medication and pain intensity, the repeated chair stands test remained significantly associated with the frequency of sexual contact (0.53; 0.01–1.05) and with satisfaction with overall sex life (1.39; 0.28–2.51). The results of this study show that problems with sexual health are highly prevalent in patients with RA. The ability to rise from a chair is associated with sexual function, independent of disease activity and pain intensity.

## Introduction

In patients with rheumatoid arthritis (RA), subjective measures such as quality of life and health satisfaction have been recognised as very important health outcomes [1]. Sexual health is one of the strongest determinants of general health and quality of life [2], especially in patients affected by diseases associated with chronic pain [3]. In patients with RA, sexuality has not been comprehensively examined, and it is often a neglected aspect of quality of life [4].

Compared to the general population, sexual health, which includes sexual function, sexual activity and sexual relationships, is often significantly affected in patients with RA [5–8]. In female RA patients, many aspects of sexual function, such as desire, arousal, lubrication, orgasm and satisfaction, are often affected [7], while male RA patients often suffer from impaired sexual desire [9] and erectile dysfunction [10].

Factors that have been found to explain the impairment in sexual health in RA patients include pain intensity, disease activity, physical capacity and disability, joint stiffness, fatigue, mental conditions and the use of certain drugs [4, 11, 12]. Additionally, co-morbidities such as diabetes mellitus or depression, as well as treatment with medication such as antihypertensives, beta blockers or psychoactive substances can cause sexual dysfunction [13].

In patients with RA, physical fitness is often impaired. Loss of muscle mass and muscle strength is common in patients with RA and is proportional to disease activity [14]. This loss of muscle strength has been shown to be associated with a higher grade of disability and a poorer quality of life [15, 16]. Sexual activity may be considered as a type of physical exercise with energy expenditure that has been classified as moderate to vigorous physical activity [17]. Therefore, a certain level of physical fitness is required, which is often limited by the disease in RA patients.

The association between fitness parameters such as muscle strength and physical performance with sexual health has rarely been examined, particularly for RA patients. In the general population, the association between sexual function (especially sexual arousal and orgasm) and muscle strength of the pelvic floor in women has been previously examined [18, 19]. In men, the association between muscle strength and sexual function, especially sexual erection and libido, is usually explained by testosterone level [20].

It was the aim of this study to examine sexual health in male and female patients with RA, to analyse the factors associated with sexual health and to explore if there is an association between sexual function and physical fitness, operationalised by muscle strength in the upper and lower extremities and physical performance.

## Methods

The study is part of a larger monocentric cross-sectional study with the primary aim of assessing workability in patients with RA. Methods used in the whole study have been published in detail separately [21]. Participants were recruited from November 2015 until August 2016 at the outpatient clinic of a hospital department with a focus on rheumatic diseases as they waited for their appointment. Inclusion criteria were: (1) age between 18 and 65 years; (2) RA according to the 2010 EULAR classification for seropositive RA [22]; (3) sufficient knowledge of the German or English language to understand the research assistant’s advice with regard to physical measurements and good knowledge of one language the questionnaires were provided in (German, English, Serbo-Croatian, Turkish). The following exclusion criteria were applied: (1) no informed consent; (2) unable to follow advice with regard to physical measurements and understand interview questions; (3) severe comorbidities, e.g. malignant diseases, severe cardio-vascular or mental illnesses, or mental or physical disabilities.

Sexual health was assessed with a self-administered questionnaire individually designed for this study. This questionnaire consisted of two sections addressing the main problems with sexual health in patients with RA, difficulties in performing intercourse and problems in sexual drive [4, 9]. The following questions were asked:

1. How often do you experience difficulties in sexual intercourse? Please enter a number between 1 and 10 (1 = never, 10 = always).
2. In case you experience difficulties in sexual intercourse, are these difficulties mainly due to: (please tick one or more boxes) “stiffness”, “pain”, “fatigue”, “arousal/erection problems”, “body image problems” or “other”.
3. How is your desire/wish to engage in sexual intercourse (with partner or other persons)? Please enter a number between 1 and 10 (1 = less than once per month, 10 = several times a day).
4. How often have you (approximately) had sexual contact during the last month? Please enter a number between 1 and 10 (1 = less than once per month, 10 = several times a day).
5. How satisfied do you feel with your overall sex life? Please enter a number between 1 and 10 (1 = not satisfied at all, 10 = completely satisfied).

For the measurement of maximum handgrip strength and maximum knee extensor strength, these were measured on both sides. Handgrip strength was measured with a portable Jamar hydraulic hand dynamometer (Patterson Medical). According to a standardised procedure [23], patients were instructed to sit upright with their upper arm adducted and the elbow flexed at 90°. Knee extensor strength was assessed isometrically with a dynamometer in a standardised procedure [24]. During the test, patients were instructed to sit straight with 90° flexion in the hips. Hip and thigh were fixed, arms were crossed, and ankles were fixed in a flexed position to the dynamometer. A load cell (Chatillon, Ametek Inc) was mounted on the ankle via a length-adjustable cord. For all four extremities, strength was assessed three times with a break of 2 min between each try. The highest values were used for the analyses.

Physical performance was assessed with the Short Physical Performance Battery (SPPB) [25]. This test consists of five tasks: one for gait speed, a repeated chair stands test and three tasks assessing balance skills. For each category, patients can score 4 points, with 0 representing inability to complete the test and 4 representing the highest level of performance. A score for each of the three domains and a summary score of the total SPPB ranging from 0 (worst performance) to 12 (best performance) were used for the analyses.

Pain intensity was assessed with a visual analogue scale (VAS) [26], where 1 was no pain at all and 10 the maximum pain imaginable. Disease activity was measured by a medical doctor using the Clinical Disease Activity Index (CDAI), a measurement of disease activity in seropositive RA [27]. The following formula was used: CDAI = SJC (28) + TJC (28) + PGA + EGA. SJC is the swollen 28-joint count (shoulders, elbows, wrists, metacarpophalangeal joints, proximal interphalangeal joints, including thumb interphalangeal joint, knees); TJC-28 is the tender 28-joint count; PGA is the patient’s self-assessment of the overall RA disease activity on a scale of 1–10, where 10 is the maximum activity; and EGA is the evaluator’s assessment of the overall RA disease activity.

Demographic data such as gender, age, language of the questionnaire filled in, marital status, education level and employment status were recorded. In addition, clinical data, including drug therapy and presence of comorbidities, were assessed. Co-medication were recorded from the patients’ charts. For the analysis, angiotensin converting enzyme (ACE) inhibitors, beta blockers, and psychoactive substances (antidepressants, benzodiazepines, and neuroleptic drugs) were regarded as relevant parameters. Disease duration in months was assessed by the study assistant using the question, “When was RA first diagnosed?”.

Sample size calculation was performed according to the main study objective, which was to determine the difference in work ability between patients with high versus low disease activity. We estimated the percentage of good work ability to be 40% in patients with high disease activity and 80% in those with low disease activity, and that 70% of the included participants would have a low disease activity. With an alpha risk of 0.05 and a beta risk of 0.2 accepted in a 2-sided test, 71 patients needed to be included. With an assumed 30% rate of loss due to noncompliance during physical tests, lack of understanding, or refusal to participate, a total of 100 included patients was planned to be included.

To determine the data distribution for the categorical variables, the Kolmogorov–Smirnov test and histogram analysis were applied. In the case of a normal distribution, mean and standard deviation (SD) are presented and the student’s *t* test was performed. In the case of a skewed distribution, median and interquartile range (IQR) are presented and the Mann–Whitney *U* test was performed. Categorical variables are presented with *N* and percentages and the Chi^2^ test, or in the case of *N* < 5 in any subgroup, the Fisher’s exact test was performed. Characteristics of the subjects and differences between those who responded to the questions about sexual functioning (filled in at least one of the three questions: how often they experienced desire to engage in sexual intercourse, how often they actually had sexual contact or how satisfied they were with their overall sex life) and those who did not respond are shown in Table 1. Correlations between socio-demographic, clinical and fitness parameters with desire for sexual intercourse, frequency of sexual contact, and satisfaction with overall sex life are shown with two-sided Pearson’s correlation coefficients or Spearman’s correlation coefficients (*r*) in the case of a skewed distribution or categorical variable in Table 2. Furthermore, we performed linear regression analyses with the scores of desire for sexual intercourse (Table 3), frequency of sexual contact (Table 4) and satisfaction with overall sex life (Table 5) as the dependent variables. Maximum strength in the four measured sites, total SPPB score and the score in each of the three SPPB domains were the independent variables. For each fitness parameter, separate regression models were calculated. We calculated crude models (model I) and stepwise adjusted for possible confounders: age and gender (model II); additional disease activity (model III); additional co-morbidities and the treatment with ACE inhibitors, beta blockers, and psychoactive substances (model IV); additional pain intensity (model V). Results are presented as unstandardised B coefficients and 95% confidence intervals. Statistics were calculated with SPSS version 24 (IBM Corp), and *p* values of < 0.05 were considered statistically significant.

**Table 1:** Socio-demographic and clinical characteristics among subjects who participated vs. those who did not participate in the questionnaire on sexual functioning

	Subjects who participated in the questionnaire on sexual functioning (*N* = 54)	Subjects who did not participate in the questionnaire on sexual functioning (*N* = 46)	*P*
Age in years; mean (SD)	47.8 (10.6)	53.7 (7.5)	0.001
Female; *N* (%)	33 (61)	33 (72)	0.263
Married, firm relationship; *N* (%)	40 (74)	31 (67)	0.463
German questionnaire; *N* (%)	52 (96)	37 (80)	< 0.001
English questionnaire; *N* (%)	2 (4)	–	
Serbo-Croatian questionnaire; *N* (%)	–	5 (11)	
Turkish questionnaire; *N* (%)	–	4 (9)	
Primary education; *N* (%)	6 (11)	12 (26)	0.091
Secondary education; *N* (%)	40 (74)	31 (67)	
Tertiary education; *N* (%)	8 (15)	3 (7)	
Currently employed	37 (69)	22 (48)	0.036
Disease duration in months; median (IQR)	60 (24–96)	90 (48–255)	0.006
Therapy with DMARDs; *N* (%)	44 (82)	36 (78)	0.688
Therapy with biologics; *N* (%)	23 (43)	21 (46)	0.759
Therapy with corticosteroids; *N* (%)	11 (20)	6 (13)	0.331
Therapy with NSAIDs/pain killers; *N* (%)	7 (13)	8 (17)	0.537
Presence of comorbidities; *N* (%)	31 (57)	34 (74)	0.085
Therapy with ACE inhibitors	9 (17)	9 (20)	0.707
Therapy with beta blockers	8 (15)	6 (13)	0.799
Therapy with psychoactive drugs	4 (7)	8(17)	0.126
Pain intensity in points VAS; mean (SD)	3.2 (1.8)	4.2 (2.2)	0.015
Disease activity in points CDAI; median (IQR)	6 (0–411.5)	9 (2–14)	0.317
Max. handgrip strength right in kg; mean (SD)	33.5 (15.8)	26.4 (14.9)	< 0.001
Max. handgrip strength left in kg; mean (SD)	31.6 (14.2)	25.2 (13.4)	< 0.001
Max. knee extensor strength right in kg; mean (SD)	42.8 (17.2)	30.1 (15.2)	0.024
Max. knee extensor strength left in kg; mean (SD)	41.5 (16.0)	29.2 (15.4)	0.024
Physical performance in points SPPB; median (IQR)	12 (11–12)	11 (9–12)	0.002
Physical performance in points SPPB repeated chair stands; median (IQR)	4 (3.75–4)	3 (1.75–4)	0.002
Physical performance in points SPPB balance test; median (IQR)	4 (4–4)	4 (3.75–4)	0.036
Physical performance in points SPPB walking test; median (IQR)	4 (4–4)	4 (4–4)	0.002

*SD* standard deviation, *IQR* interquartile range, *DMARD* disease-modifying antirheumatic drug, *NSAIDs* non-steroidal anti-inflammatory drug, *ACE* angiotensin converting enzyme, *VAS* visual analogue scale, *CDAI* clinical disease activity index, *SPPB* short physical performance battery

**Table 2:** Correlation of socio-demographic, clinical and physical fitness parameters with parameters of sexual health

	Desire for sexual intercourse		Frequency of sexual contact		Satisfaction with overall sex life	
	*r*	*P*	*r*	*P*	*r*	*P*
Age	− 0.196	0.176	− 0.081	0.580	− 0.094	0.535
Gender	− 0.440	0.002	− 0.294	0.040	− 0.027	0.857
Pain intensity (VAS)	− 0.165	0.256	− 0.112	0.442	− 0.304	0.040
Disease activity (CDAI)	− 0.149	0.311	0.042	0.776	− 0.178	0.242
Therapy with DMARDs	− 0.126	0.390	− 0.118	0.421	− 0.156	0.300
Therapy with biologics	− 0.005	0.976	− 0.166	0.026	0.289	0.051
Therapy with corticosteroids	− 0.131	0.368	0.002	0.989	− 0.034	0.822
Therapy with NSAIDs/pain killers	− 0.029	0.841	− 0.069	0.636	− 0.220	0.142
Comorbidity	− 0.006	0.968	0.105	0.471	0.237	0.113
Therapy with ACE inhibitors	− 0.086	0.558	− 0.139	0.342	− 0.111	0.463
Therapy with beta blockers	− 0.381	0.007	− 0.302	0.162	− 0.311	0.035
Therapy with psychoactive substances	− 0.326	0.022	− 0.310	0.030	− 0.381	0.009
Max. handgrip strength right	0.439	0.002	0.336	0.018	0.330	0.025
Max. handgrip strength left	0.449	0.001	0.288	0.047	0.206	0.170
Max. knee extensor strength right	0.491	< 0.001	0.451	0.001	0.376	0.012
Max. knee extensor strength left	0.439	0.002	0.319	0.026	0.249	0.095
Physical performance (SPPB total)	0.197	0.175	0.237	0.100	0.484	0.001
SPPB repeated chair stands test	0.221	0.126	0.248	0.086	0.433	0.003
SPPB balance test	− 0.055	0.709	0.025	0.864	0.217	0.147
SPPB walking test	0.224	0.121	0.224	0.123	0.130	0.389

*r* Pearson’s correlation coefficient (pain intensity, strength scores) or Spearman’s correlation coefficient (gender, disease activity, medication, comorbidity, co-medication, SPPB scores), *VAS* visual analogue scale, *CDAI* clinical disease activity index, *DMARD* disease-modifying antirheumatic drug, *NSAIDs* non-steroidal anti-inflammatory drug, *ACE* angiotensin converting enzyme, *SPPB* short physical performance battery

**Table 3:** Association of parameters of physical fitness with the desire for sexual intercourse (dependent variable) in patients with RA; results of the stepwise adjusted linear regression analyses

	Model I		Model II		Model III		Model IV		Model V	
	RC	95% CI	RC	95% CI	RC	95% CI	RC	95% CI	RC	95% CI
Max. handgrip strength right	0.06**	0.03 to 0.08	0.04*	0.00 to 0.08	0.03	− 0.01 to 0.07	0.03	− 0.01 to 0.06	0.03	−0.01 to 0.07
Max. handgrip strength left	0.06*	0.02 to 0.09	0.02	− 0.01 to 0.08	0.02	− 0.03 to 0.07	0.02	− 0.03 to 0.06	0.02	−0.03 to 0.07
Max. knee extensor strength right	0.05**	0.02 to 0.08	0.03	− 0.00 to 0.06	0.03	− 0.01 to 0.06	0.02	− 0.01 to 0.05	0.02	−0.02 to 0.05
Max. knee extensor strength left	0.05*	0.02 to 0.08	0.02	− 0.02 to 0.06	0.02	− 0.02 to 0.06	0.02	− 0.02 to 0.05	0.02	− 0.02 to –0.05
SPPB total	0.39	− 0.05 to 0.84	0.50*	0.10 to 0.89	0.37	− 0.06 to 0.81	0.31	− 0.10 to 0.71	0.31	− 0.11 to 0.72
SPPB repeated chair stands	0.57*	0.04 to 1.12	0.73*	0.26 to 1.20	0.61*	0.11–1.11	0.45	− 0.04 to 0.94	0.45	− 0.05 to 0.96
SPPB balance test	− 0.32	− 1.68 to 1.04	− 0.42	− 1.63 to 0.78	− 0.64	− 1.82 to 0.53	− 0.15	− 1.29 to 1.00	− 0.15	− 1.31 to –1.01
SPPB walking test	3.01	− 0.59 to 6.61	3.70*	0.57 to 6.83	2.68	− 0.80 to 6.11	1.81	− 1.82 to 5.45	1.94	− 1.79 to 5.66

Desire for sexual intercourse was a metric variable, with 1 = less than once per month and 10 = several times a day

Model I: unadjusted

Model II: adjusted for age and gender

Model III: adjusted for age, gender and disease activity

Model IV: adjusted for age, gender, disease activity, co-morbidity, treatment with ACE inhibitors, beta blockers and psychoactive substances

Model V: adjusted for age, gender, disease activity, co-morbidity, treatment with ACE inhibitors, beta blockers, psychoactive substances and pain intensity

*RC* regression coefficient, *CI* confidence interval, *SPPB* short physical performance battery

*P < 0.05; **P < 0.001

**Table 4:** Association of parameters of physical fitness with frequency of sexual contact (dependent variable) in patients with RA; results of the stepwise adjusted linear regression analyses

	Model I		Model II		Model III		Model IV		Model V	
	RC	95% CI	RC	95% CI	RC	95% CI	RC	95% CI	RC	95% CI
Max. handgrip strength right	0.03*	0.01 to 0.06	0.03	−0.01 to 0.06	0.02	−0.02 to 0.06	0.01	−0.02 to 0.05	0.01	−0.04 to 0.05
Max. handgrip strength left	0.03*	0.00 to 0.06	0.02	−0.03 to 0.07	0.02	−0.03 to 0.07	0.01	−0.04 to 0.06	0.00	−0.05 to 0.05
Max. knee extensor strength right	0.04*	0.02 to 0.07	0.04*	0.01 to 0.07	0.03*	0.00 to 0.07	0.03	−0.01 to 0.06	0.02	−0.01 to 0.06
Max. knee extensor strength left	0.03*	0.00 to 0.06	0.02	−0.02 to 0.06	0.02	−0.02 to 0.06	0.02	−0.02 to 0.06	0.02	−0.02 to 0.05
SPPB total	0.35	−0.06 to 0.75	0.45*	0.05 to 0.86	0.47*	0.03 to 0.91	0.42*	0.00 to 0.84	0.41	−0.02 to 0.83
SPPB repeated chair stands	0.49	−0.01 to 0.99	0.64*	0.15 to 1.14	0.66*	0.13 to 1.19	0.55*	0.04 to 1.06	0.53*	0.01 to 1.05
SPPB balance test	−0.07	−1.31 to 1.12	−0.14	−1.34 to 1.06	−0.19	−1.42 to 1.04	0.29	−0.91 to 1.50	0.29	−0.92 to 1.50
SPPB walking test	2.77	−0.44 to 5.98	3.24*	0.11 to 6.38	3.29	−0.23 to 6.82	1.37	−2.61 to 5.35	1.59	−0.51 to 0.18

Frequency of sexual contact was a metric variable, with 1 = less than once per month and 10 = several times a day

Model I: unadjusted

Model II: adjusted for age and gender

Model III: adjusted for age, gender and disease activity

Model IV: adjusted for age, gender, disease activity, co-morbidity, treatment with ACE inhibitors, beta blockers and psychoactive substances

Model V: adjusted for age, gender, disease activity, co-morbidity, treatment with ACE inhibitors, beta blockers, psychoactive substances and pain intensity

*RC* regression coefficient, *CI* confidence interval, *SPPB* short physical performance battery

*P < 0.05; **P < 0.001

**Table 5:** Association of parameters of physical fitness with satisfaction with overall sex life (dependent variable) in patients with RA; results of the stepwise adjusted linear regression analyses

	Model I		Model II		Model III		Model IV		Model V	
	RC	95% CI	RC	95% CI	RC	95% CI	RC	95% CI	RC	95% CI
Max. handgrip strength right	0.06*	0.01 to 0.12	0.09*	0.02 to 0.16	0.09*	0.01 to 0.16	0.07	−0.01 to 0.14	0.06	−0.02 to 0.15
Max. handgrip strength left	0.04	−0.02 to 0.10	0.06	−0.02 to 0.15	0.05	−0.04 to 0.14	0.04	−0.06 to 0.13	0.02	−0.07 to 0.12
Max. knee extensor strength right	0.07*	0.02 to 0.12	0.08*	0.02 to 0.14	0.07*	0.01 to 0.13	0.06	0.00 to 0.12	0.05	−0.02 to 0.12
Max. knee extensor strength left	0.05	−0.01 to 0.12	0.06	−0.01 to 0.14	0.06	−0.02 to 0.14	0.06	−0.02 to 0.13	0.05	−0.02 to 0.13
SPPB total	1.62**	0.83 to 2.40	1.73**	0.90 to 2.56	1.70**	0.78 to 2.62	1.41*	0.46 to 2.37	1.35	−0.34 to 2.36
SPPB repeated chair stands	1.71**	0.82 to 2.59	1.86**	0.94 to 2.80	1.79*	0.77 to 2.80	1.47*	0.41 to 2.52	1.39*	0.28 to 2.51
SPPB balance test	2.60	−0.96 to 6.15	2.52	−1.19 to 6.23	2.24	−1.51 to 5.98	2.77	−0.95 to 6.49	2.51	−1.27 to 6.28
SPPB walking test	2.82	−3.28 to 8.92	2.93	−3.44 to 9.31	1.17	−5.96 to 8.29	−1.81	−9.42 to 5.81	−1.00	−8.77 to 6.77

Satisfaction with overall sex life was a metric variable, with 1 = not satisfied at all and 10 = completely satisfied

Model I: unadjusted

Model II: adjusted for age and gender

Model III: adjusted for age, gender and disease activity

Model IV: adjusted for age, gender, disease activity, co-morbidity, treatment with ACE inhibitors, beta blockers and psychoactive substances

Model V: adjusted for age, gender, disease activity, co-morbidity, treatment with ACE inhibitors, beta blockers, psychoactive substances and pain intensity

*RC* regression coefficient, *CI* confidence interval, *SPPB* short physical performance battery

*P < 0.05; **P < 0.001

## Results

A total of 140 consecutive patients with seropositive RA who fulfilled the inclusion criteria were invited to participate in the study. Out of those, 100 patients were included in the study. Thirteen patients refused to participate due to lack of interest, 6 refused due to lack of time, 4 had to be excluded due to language problems, and 17 participants initially agreed to participate, but did not come to the agreed appointment for strength measurements. Out of the 100 participants of the study, 54 also filled in the questionnaire on sexual health. As reasons for refusing to answer questions on sexuality, cultural/religious reasons were most often mentioned, especially in patients with migration background. Other patients explained that they regarded those questions as too intimate or that sexuality did not play a role in their life anymore. Socio-demographic and clinical characteristics of the respondents (and of those who did not respond) are shown in Table 1. Mean age of the respondents was 48 years, and two-third were female. Three-fourth had secondary education and most participants were currently employed. The median duration of the disease was 5 years. Most patients had received therapy with disease-modifying anti-rheumatic drugs and one-half received biologics. One-half also had comorbidities. A minority of patients had ACE inhibitors, beta blockers or psychoactive substances as co-medication. Mean pain intensity was 3 on the 10-point VAS scale. Disease activity (CDAI score) was at a mean of 8 points and thus in the “low disease activity” range. Mean handgrip strength was 34 kg right and 32 kg left, and mean knee extensor strength was 43 kg right and 42 kg left. Physical performance showed a mean of 11 points (out of a max. 12 points) (Table 1).

Patients who did not answer the questions about sexual health were significantly older, and less often employed. All of the subjects who filled in the questionnaire in Serbo-Croatian or in Turkish were among those who did not answer the questions about sexual health. Patients who did not respond had a significantly longer median disease duration and a higher mean pain intensity. All dimensions of physical fitness (handgrip and knee extensor strength, and physical performance) were significantly better in subjects who replied to the questionnaire on sexual health (Table 1).

Out of the 54 patients who filled in the questionnaire about sexual function, 57.7% reported, at least, sometimes having difficulty in sexual intercourse. Reasons for difficulty in sexual intercourse were joint stiffness (27.8%), fatigue (24.1%), pain (18.5%), body image problems (9.3%), arousal/erection problems (7.4%) and other reasons (5.6%).

Forty-nine subjects answered the question about the desire to engage in sexual intercourse. On a scale with 1 meaning “less than once per month” and 10 “several times a day”, the patients answered with a mean (SD) score of 4.0 (1.8), with a range from 1 to 8. Forty-nine patients also answered the question of how often they had sexual contact during the last month. On a scale where 1 was “less than once per month” and 10 “several times a day”, the mean (SD) score was 3.7 (1.6), with a range from 1 to 6. Forty-six patients answered how satisfied they were with their overall sex life. On a scale where 1 indicated “not satisfied at all” and 10 “completely satisfied”, the mean (SD) score was 5.8 (3.0), with a range from 1 to 10.

Table 2 shows the correlation between socio-demographic, clinical and physical fitness parameters with the parameter of sexual health. Male gender significantly correlated with the desire for sexual intercourse and the frequency of sexual contact. Pain intensity inversely correlated with satisfaction with overall sex life. Handgrip strength and knee extensor strength correlated with all three parameters of sexual health. The SPPB total score and the SPPB repeated chair stands test score correlated with satisfaction with overall sex life. Furthermore, there was a weak but significant negative correlation between medication with biologics and frequency of sexual contact, as well as between beta blockers and desire for sexual intercourse and satisfaction with overall sex life, and between psychoactive substances and all three measures of sexual health. Age and disease activity correlated only weakly and non-significantly inversely with the parameters of sexual health.

In the linear regression analysis, muscle strength in all four limbs and the results of the repeated chair stands test were significantly associated with the desire for sexual intercourse. After adjusting for age and gender, hand grip strength right, the SPPB total score, the repeated chair stands test and the walking test remained significant. After adjusting additionally for disease activity, only the repeated chair stands test remained significantly associated with the desire for sexual intercourse. Adjusting additionally for co-morbidity, co-medication, and pain intensity yielded in a loss of significance in the association between desire for sexual intercourse and parameters of physical fitness (Table 3).

Similarly, strength in all four limbs was associated with the frequency of sexual intercourse in the unadjusted model. Adjusting for age and gender yielded a significant association between knee extensor strength left, the SPPB total score, the repeated chair stands test and the walking test. After adjusting additionally for disease activity, co-morbidity, co-medication, and pain intensity, only the repeated chair stands test remained significant (Table 4).

Handgrip strength right, knee extensor strength right, the SPPB total score and the repeated chair stands score were significantly associated with the satisfaction with overall sex life in the unadjusted model. Adjusting for age, gender and disease activity did not change those associations. After adjusting additionally for co-morbidity, co-medication, and pain intensity, only the SPPB repeated chair stands test score remained significantly associated with the satisfaction with overall sex life (Table 5).

## Discussion

Our findings showed that more than half of the respondents of patients with RA had problems with sexual intercourse. Besides gender, pain intensity, medication, and co-medication, the parameters of physical fitness (muscle strength in the extremities and physical performance) correlated with sexual performance and sexual satisfaction. The association between physical fitness parameters and parameters of sexual health clearly weakened when adjusted for disease activity, pain intensity, comorbidities, and co-medication. Therefore, those parameters seemed to contribute to the explanation of the found association; however, some parameters of physical fitness remained significantly associated with sexual function in the fully adjusted model.

According to the existent scientific literature, the proportion of RA patients with sexual problems ranges from 31 to 76% [4]. The proportion of 58% in our study reporting difficulties with sexual intercourse is therefore in the middle of this range. It has to be taken into account, however, that the patients in our study were relatively young (< 65 years as inclusion criteria and mean age of 48 years), and problems with sexual health increase with age. In addition, the reasons for sexual problems in our study (joint stiffness, fatigue, pain, body image problems and arousal/erection problems) and the association of sexual problems with pain intensity and disease activity are similar to findings reported in previous studies [4, 7, 9–12].

Interestingly, we found an association between sexual parameters and parameters of physical fitness. Handgrip strength and knee extensor strength, especially on the right side, as well as physical performance, especially the repeated chair stands test, were associated with the desire to engage in sexual intercourse, with the frequency of sexual contact and with the satisfaction with overall sex life. The associations were similar for all three of the included parameters of sexual health, but were slightly stronger regarding satisfaction with overall sex life (according to the magnitude of the regression coefficient). Adjusting for age and gender had little effect on altering these associations, additionally adjusting for disease activity only marginally reduced these associations and additionally adjusting for comorbidities, co-medication, and pain intensity clearly weakened this association. This can be interpreted as disease activity only marginally mediates and pain intensity together with comorbidities and co-medication strongly mediates the association between physical fitness and sexual health in RA patients. Still, there was a significant independent association between the repeated chair stands test and the frequency of sexual contact, and the satisfaction with overall sex life, in the fully adjusted model.

As always in cross-sectional studies, no causal conclusions can be drawn from the associations we found. Explanations for the found association could be: (1) that physical fitness contributes to better sexual health; (2) that better sexual health contributes to better physical fitness; or that (3) sexual health and physical fitness are both influenced by a third factor.

What gives us a hint in favour that physical fitness contributes to better sexual health is the fact that, in our study, the strongest association was found for the repeated chair stands test and sexual health. Sexual intercourse is moderate to vigorous exercise [17], and might require the same physical prerequisites that are also necessary for the repeated chair stands test. In addition, as physical performance is often reduced in patients with RA [28], these patients might be especially prone to the consequences of this loss. This would explain the strong association between the chair stands test score and sexual health in RA patients.

It could also be asserted that sexual activity through physical training leads to better physical performance. It has been shown that physical training has many positive effects in patients with RA [29]. This could also contribute to the explanation of why physical performance is associated with sexual health in RA patients.

Finally, a third factor might explain the association between physical fitness and sexual function in RA patients. Clearly, disease activity and pain intensity could be such confounding factors, as adjusting for them weakened the association between physical fitness and sexual health in our study. Disease activity and pain intensity were found to be associated with sexual function in RA patients in previous studies [4, 11]. Disease activity and pain are also associated with physical fitness in RA patients, especially with muscular strength [14, 30]. It has been argued that loss in muscle mass and muscle strength in RA patients are consequences of chronic inflammation and the catabolic effect of inflammatory cytokines [31, 32]. Chronic inflammation, on the other hand, has been show to affect sexual function in both men [33] and women [34]. In addition, in men, low testosterone levels as a consequence of chronic inflammation might be important, since this leads to loss of muscle mass and strength as well as to a decline in sexual function [35, 36]. In women, general loss in muscle strength also includes loss of muscle strength of the pelvic floor [18, 19], and thus can explain problems with sexual health. Other possible confounders include joint stiffness and fatigue, which were most often reported as causes for problems with sexual intercourse in our study, and these factors could also explain problems in physical fitness, e.g. in the repeated chair stands test.

There are several implications of the findings for clinical routine that should be stressed: first, we cannot prove that improving physical fitness in RA patients will also improve sexual function and sexual satisfaction. However, several studies have shown the beneficial effects of physical training in RA patients [29, 37], and physical training should, therefore, be implemented in RA patients for many different reasons. In addition, the effect of physical training on chronic inflammation has been shown in several studies [38, 39], and this could, in addition to many other benefits, also improve sexual function. Furthermore, our results, in line with other studies, show how frequent sexual problems are in patients with RA, and it is, therefore, necessary to address sexual health and actively ascertain sexual function in RA patients. Open communication about sexuality with RA patients can be regarded as the first step to improve the situation, and the treatment will depend on the specific problem [4].

Some possible limitations of our study have to be mentioned: first, the relatively small sample size may lead to possible underpowerment of the statistical analyses. In addition, the response rate of 54% is quite low. The patients’ characteristics between those who responded to the questions about sexual health and those who did not also differed significantly. The non-responders were older, had a longer disease duration, a higher pain intensity and lower physical fitness. Since all these factors are associated with a higher probability of sexual problems, our findings are likely to underestimate the real problem with sexual health in RA patients. Shame of talking about sexuality might be the main reason for the low response rate, which might also be a religious or cultural issue, since the non-responders significantly more often used the questionnaire of another language than the responders. Cultural and religious issues in talking about sexuality in the medical care setting have also been reported in other studies [40]. Furthermore, it has to be mentioned that we did not use a validated instrument to assess sexual health.

In conclusion, our study shows that problems with sexual health are highly prevalent in patients with RA. Most frequently mentioned are sexual problems due to joint stiffness, fatigue and pain. Factors associated with sexual health include, gender, pain intensity and also factors of physical fitness such as muscle strength and physical performance. After controlling for disease activity and pain intensity, the ability to rise from a chair remains as an independent factor associated with sexual function.
